# Supplementary material for: Domestic dogs maintain clinical, nutritional, and hematological health outcomes when fed a commercial plant-based diet for a year
Source: PLoS One. 2024 Apr 16;19(4):e0298942. doi: 10.1371/journal.pone.0298942 (PMC11020905; doi:10.1371/journal.pone.0298942)
Supplement: S1 Table — Values reflect median (minimum—maximum). (DOCX) [file pone.0298942.s001.docx]

**S1 TABLE**. Complete blood count and blood chemistry analyses in dogs consuming meat-based diets (baseline) versus plant-based nutrition (6 and 12 months). Values reflect median (minimum - maximum).

CBC values remained within normal reference intervals. In one dog, the total leukocyte count was within the normal interval, but the neutrophil count (2.5 K/μL) was below the lower end of the reference interval at 6 months, which normalized at 12 months. Standard blood chemistry values all remained within clinically unremarkable reference intervals. One dog had a borderline low phosphorous level (2.2 mg/dL) at the endpoint (12 months) in combination with a normal vitamin D level (237 nmol/L). The sodium level (130 mmol/L) measured below the minimum value of the reference interval at baseline in one dog, which normalized at 6 and 12 months. The chloride level was borderline low (108 mmol/L) in one dog at the endpoint. The albumin level (4.3 g/dL) measured above the maximum value of the reference interval in one dog at endpoint, while total protein remained within the normal reference interval. GGT (gamma-glutamyl transferase) baseline levels were above the maximum value of the reference interval in two dogs (13 and 19 U/L), which had normalized at 6 and 12 months. The bilirubin level (1.2 mg/dL) was mildly elevated at baseline in one dog, which normalized at 6 and 12 months. Osmolality measured below (268 mmol/kg) and above (313 mmol/kg) the reference interval in two dogs at baseline, but normalized at 6 and 12 months.

| **Parameter** | **Unit** | **Baseline** | **6 months** | **12 months** | **P-value**  **(Friedman)** | **P-value**  **(Wilcoxon)** | **Reference interval**  **(IDEXX)** |
| --- | --- | --- | --- | --- | --- | --- | --- |
| *CBC* |  |  |  |  |  |  |  |
| Erythrocytes | M/μL | 7.6 (6.8-8.5) | 7.9 (6.8-8.7) | 8.0 (7.4-8.9) | 0.03 | 0.003 | 5.65-8.87 |
| Hematocrit | % | 52.0 (46.0-58.1) | 53.1 (47.5-58.4) | 54.1 (48.1-60.7) | 0.002 | 0.007 | 37.3-61.7 |
| Hemoglobin | g/dL | 19.1 (16.6-20.5) | 19.2 (17.3-20.7) | 19.6 (17.9-21.2) | 0.003 | 0.002 | 13.1-20.5 |
| Leukocytes | K/μL | 7.3 (5.2-11.9) | 7.6 (4.8-13.3) | 7.0 (5.0-12.1) | 0.59 | 0.73 | 5.05-16.76 |
| Neutrophils | K/μL | 4.7 (3.6-9.1) | 5.2 (2.5-10.5) | 4.6 (3.4-8.8) | 0.89 | 0.56 | 2.95-11.64 |
| Lymphocytes | K/μL | 1.7 (0.9-2.2) | 1.8 (1.2-3.1) | 1.7 (1.0-2.1) | 0.30 | 0.22 | 1.05-5.10 |
| Monocytes | K/μL | 0.4 (0.2-0.9) | 0.4 (0.2-0.8) | 0.4 (0.3-0.9) | 0.69 | 0.24 | 0.16-1.12 |
| Eosinophils | K/μL | 0.4 (0.3-1.0) | 0.4 (0.2-1.0) | 0.5 (0.3-1.2) | 0.62 | 0.35 | 0.06-1.23 |
| Basophils | K/μL | 0.01 (0.00-0.09) | 0.01 (0.00-0.08) | 0.02 (0.00-0.06) | 0.69 | > 0.99 | 0.00-0.10 |
| Platelets | K/μL | 202 (144-322) | 232 (140-400) | 214 (185-318) | 0.34 | 0.21 | 148-484 |
| *Chemistry* |  |  |  |  |  |  |  |
| Glucose | mg/dL | 99 (76-109) | 99 (75-109) | 107 (85-117) | 0.01 | < 0.001 | 74-143 |
| Creatinine | mg/dL | 1.0 (0.6-1.4) | 1.1 (0.8-1.4) | 1.1 (0.7-1.3) | 0.14 | 0.13 | 0.5-1.8 |
| BUN | mg/dL | 20 (9-23) | 15 (6-23) | 19 (6-27) | 0.09 | 0.77 | 7-27 |
| Phosphorus | mg/dL | 4.4 (3.2-5.8) | 4.0 (2.5-6.0) | 3.9 (2.2-4.8) | 0.08 | 0.01 | 2.5-6.8 |
| Calcium | mg/dL | 10.2 (9.8-10.6) | 10.1 (9.6-10.6) | 10.2 (9.1-10.5) | 0.21 | 0.36 | 7.9-12.0 |
| Sodium | mmol/L | 153 (130-158) | 153 (146-156) | 150 (145-152) | 0.002 | 0.01 | 144-160 |
| Potassium | mmol/L | 4.2 (3.6-5.0) | 4.4 (3.8-5.5) | 4.6 (4.2-5.5) | < 0.001 | 0.001 | 3.5-5.8 |
| Chloride | mmol/L | 117 (112-125) | 115 (110-119) | 113 (108-116) | 0.03 | 0.004 | 109-122 |
| Total Protein | g/dL | 6.7 (6.0-7.4) | 6.9 (6.1-7.7) | 6.9 (6.1-7.4) | 0.27 | 0.97 | 5.2-8.2 |
| Albumin | g/dL | 3.1 (2.7-3.5) | 3.4 (3.0-3.9) | 3.6 (3.1-4.3) | 0.002 | 0.003 | 2.3-4.0 |
| ALT | U/L | 97 (30-129) | 61 (22-115) | 51 (21-357) | 0.06 | 0.12 | 10-125 |
| ALP | U/L | 61 (13-222) | 44 (22-222) | 40 (10-192) | 0.15 | 0.05 | 23-212 |
| GGT | U/L | 3 (0-19) | 1 (0-7) | 3 (0-6) | 0.003 | 0.62 | 0-11 |
| Bilirubin | mg/dL | 0.4 (0.1-1.2) | 0.4 (0.2-0.6) | 0.2 (0.1-0.5) | 0.02 | 0.006 | 0.0-0.9 |
| Cholesterol | mg/dL | 178 (121-290) | 210 (111-288) | 193 (131-280) | 0.52 | 0.40 | 110-320 |
| Osmolality | mmol/kg | 303 (268-313) | 303 (289-310) | 301 (291-305) | 0.24 | 0.12 | 290-310 |
| T4 | μg/dL | 1.7 (0.9-2.6) | 1.5 (1.0-3.7) | 1.8 (1.1-3.7) | 0.42 | 0.32 | 1.0-4.0 |
