# Supplementary material for: Domestic dogs maintain clinical, nutritional, and hematological health outcomes when fed a commercial plant-based diet for a year
Source: PLoS One. 2024 Apr 16;19(4):e0298942. doi: 10.1371/journal.pone.0298942 (PMC11020905; doi:10.1371/journal.pone.0298942)
Supplement: S2 Table — Values refer to median (minimum—maximum). N/A: not applicable (since each row has zero difference, which precludes calculation of a paired test). Semi-quantitative UA parameters were tabulated as ‘0’ (normal/negative/not detected or <1/HPF), ‘1’ (trace), or the highest reported value (e.g., 1-5/HPF and 6-20/HPF were tabulated as ‘5’ and ‘20’, respectively), where HPF is high power field. Urine samples were collected via cystocentesis where iatrogenic microscopic hematuria is to be expected. (DOCX) [file pone.0298942.s002.docx]

**S2TABLE.** Urinalysis in dogs consuming meat-based diets (baseline) versus plant-based nutrition (6 and 12 months). Values refer to median (minimum - maximum). N/A: not applicable (since each row has zero difference, which precludes calculation of a paired test). Semi-quantitative UA parameters were tabulated as ‘0’ (normal/negative/not detected or <1/HPF), ‘1’ (trace), or the highest reported value (e.g., 1-5/HPF and 6-20/HPF were tabulated as ‘5’ and ‘20’, respectively), where HPF is high power field. Urine samples were collected via cystocentesis where iatrogenic microscopic hematuria is to be expected.

Urine pH trended downwards and towards normal - although findings did not reach statistical significance (p = 0.05) - as levels were elevated in eight dogs at baseline (pH 8-9), four dogs at 6 months (pH 8) and two dogs at endpoint (pH 8). Potential crystal formation is associated with changes in urine pH. In this study, we identified a variety of crystals in the urine from a total of 60% of the dogs (9 of 15) at different time points (including 3 dogs at baseline, 3 different dogs at 6 months, and 3 different dogs at 12 months) with no identifiable pattern to the changes.

| **Parameter** | **Baseline** | **6 months** | **12 months** | **P-value**  **(Friedman)** | **P-value**  **(Wilcoxon)** | **Normal Values** |
| --- | --- | --- | --- | --- | --- | --- |
| Specific gravity | 1.044 (1.020-1.050) | 1.036 (1.015-1.050) | 1.036 (1.015-1.050) | 0.06 | 0.06 | Variable |
| Glucose | 0 (0-0) | 0 (0-100) | 0 (0-0) | 0.37 | N/A | Negative |
| Bilirubin | 0 (0-1) | 0 (0-1) | 0 (0-1) | 0.37 | 0.63 | Negative |
| Ketones | 0 (0-15) | 0 (0-15) | 0 (0-15) | 0.20 | N/A | Negative |
| pH | 8 (5-9) | 7 (5-9) | 7 (5-8) | 0.05 | 0.03 | 5.0-7.5 |
| Protein | 1 (0-30) | 1 (0-30) | 1 (0-30) | 0.61 | 0.31 | Negative |
| Heme | 0 (0-50) | 10 (0-250) | 0 (0-25) | 0.01 | 0.14 | Negative |
| Erythrocytes | 0 (0-14) | 1 (0-50) | 0 (0-4) | 0.66 | 0.26 | 0-5/HPF |
| Leukocytes | 0 (0-17) | 0 (0-9) | 0 (0-3) | 0.63 | 0.44 | 0-5/HPF |
| Casts | 0 (0-0) | 0 (0-1) | 0 (0-0) | 0.14 | N/A | None |
| Epithelial cells | 0 (0-2) | 0 (0-5) | 0 (0-2) | 0.20 | 0.63 | Variable |
| Crystals | 0 (0-20) | 0 (0-5) | 0 (0-50) | 0.72 | 0.44 | None |
| Bacteria | 0 (0-1) | 0 (0-0) | 0 (0-0) | 0.37 | > 0.99 | None |
