# Supplementary material for: Domestic dogs maintain clinical, nutritional, and hematological health outcomes when fed a commercial plant-based diet for a year
Source: PLoS One. 2024 Apr 16;19(4):e0298942. doi: 10.1371/journal.pone.0298942 (PMC11020905; doi:10.1371/journal.pone.0298942)
Supplement: S3 Table — Values refer to median (first quartile—third quartile). Reference values for first and third quartiles were provided by UCD. We requested reference intervals (minimum to maximum) from this laboratory, but were informed they were not available. (DOCX) [file pone.0298942.s003.docx]

**S3TABLE.** Nutrient analysis including plasma amino acid and serum L-carnitine concentrations in dogs consuming meat-based diets (baseline) versus plant-based nutrition (6 and 12 months). Values refer to median (first quartile - third quartile). Reference values for first and third quartiles were provided by UCD. We requested reference intervals (minimum to maximum) from this laboratory, but were informed they were not available.

When evaluating the entire data set (as shown in Figure 2), some data points were below the Q_1_ for six of the ten *essential* AAs. We initially extrapolated the reference interval (minimum to maximum) for the essential amino acid tryptophan (11 – 103 nmol/L) based on the first (Q_1_) and third (Q_3_) quartiles, and the interquartile range (IQR), as follows. If IQR = Q_3_ - Q_1_, the maximum can be calculated as (Q_3_ + 1.5 x IQR = 68 + 1.5 x 23 = 103) and the minimum as (Q_1_ – 1.5 x IQR = 45 – 1.5 x 23 = 11). We moreover used extrapolation to calculate minimum values for arginine (28), leucine (36.5), methionine (15), threonine (28.5), and valine (56.5), as shown in Figure 2.

Three of the *essential* AA levels were significantly different between baseline and endpoint, including methionine (p = 0.02), phenylalanine (p = 0.01), and tryptophan (p = 0.01). Values were either within or above the interquartile reference intervals (first quartile - third quartile) provided by UCD, and above the minimum values derived through extrapolation. The values for all three of these AAs trended upwards with higher values at endpoint compared to baseline.

Statistically significant differences were found in the levels of five *non-essential* AAs between baseline and endpoint, including alanine (p < 0.001), cystathionine (p < 0.001), glutamate (p = 0.02), glutamine (p < 0.001), serine (p = 0.03), and tyrosine (p = 0.001). All measured within or above the reference intervals and values for all five AAs exhibited an upwards trend. Another four *non-essential* AAs (cysteine, hydroxyproline, ornithine, proline) measured below the first quartile, but showed no statistically significant differences between baseline and endpoint values. Cysteine levels were low, or below detection, in most samples (which is commonly attributed to storage loss). Hydroxyproline and proline levels increased over time, while ornithine levels decreased slightly.

| **Amino acid** | **Baseline (nmol/ml)** | **6 months (nmol/ml)** | **12 months (nmol/ml)** | **P-value**  **(Friedman)** | **P-value**  **(Wilcoxon)** | **Ref. Values**  **(UC Davis)** |
| --- | --- | --- | --- | --- | --- | --- |
| *Essential* |  |  |  |  |  |  |
| Arginine | 118 (112-144) | 103 (99-109) | 127 (112-164) | < 0.001 | 0.72 | 85-123 |
| Histidine | 84 (79-89) | 86 (84-88) | 94 (88-105) | 0.01 | 0.07 | 60-80 |
| Isoleucine | 84 (73-90) | 64 (58-68) | 75 (61-82) | 0.001 | 0.22 | 40-57 |
| Leucine | 134 (114-154) | 125 (111-129) | 138 (127-179) | 0.03 | 0.12 | 95-134 |
| Lysine | 149 (140-160) | 156 (140-169) | 167 (148-238) | 0.13 | 0.09 | 94-159 |
| Methionine | 51 (48-56) | 51 (49-53) | 68 (51-78) | 0.01 | 0.02 | 45-65 |
| Phenylalanine | 61 (59-64) | 72 (66-73) | 72 (66-79) | 0.002 | 0.01 | 39-52 |
| Threonine | 172 (136-298) | 162 (151-185) | 226 (207-246) | 0.03 | 0.60 | 138-211 |
| Tryptophan | 36 (24-42) | 35 (34-42) | 52 (39-75) | 0.01 | 0.01 | 45-68 |
| Valine | 173 (137-201) | 155 (145-158) | 179 (157-214) | 0.04 | 0.32 | 130-179 |
| *Non-essential* |  |  |  |  |  |  |
| Alanine | 396 (352-432) | 562 (551-588) | 578 (533-651) | < 0.001 | < 0.001 | 320-455 |
| Asparagine | 63 (50-73) | 45 (42-49) | 55 (47-96) | 0.01 | 0.95 | 30-49 |
| Aspartate | 11 (9-13) | 9 (6-9) | 10 (9-15) | 0.02 | 0.79 | 6-8 |
| Butyrate | 22 (18-24) | 32 (25-34) | 17 (15-27) | 0.06 | 0.57 | ND |
| Citrulline | 52 (45-56) | 36 (35-44) | 53 (34-65) | 0.04 | 0.92 | 27-50 |
| Cystathionine | 5 (5-6) | 8 (7-9) | 8 (8-10) | < 0.001 | < 0.001 | ND |
| Cysteine | 0 (0-1) | 0 (0-4) | 1 (0-1) | 0.32 | 0.34 | 36-53 |
| Glutamate | 72 (59-84) | 97 (74-124) | 91 (75-106) | 0.01 | 0.02 | 15-26 |
| Glutamine | 564 (539-590) | 643 (626-683) | 735 (693-773) | < 0.001 | < 0.001 | 417-569 |
| Glycine | 201 (185-243) | 185 (174-187) | 241 (195-351) | 0.01 | 0.38 | 207-310 |
| Hydroxyproline | 21 (16-33) | 12 (9-18) | 29 (18-51) | 0.01 | 0.22 | 44-78 |
| Methylhistidine1 | 11 (9-14) | 6 (4-9) | 8 (3-13) | 0.01 | 0.08 | ND |
| Methylhistidine3 | 5 (4-7) | 2 (1-4) | 5 (3-6) | 0.01 | 0.45 | ND |
| Ornithine | 19 (9-27) | 14 (12-16) | 15 (13-18) | 0.62 | 0.19 | 23-43 |
| Proline | 161 (112-201) | 147 (129-172) | 202 (161-206) | 0.08 | 0.15 | 174-304 |
| Serine | 121 (114-128) | 121 (112-125) | 137 (122-161) | 0.01 | 0.03 | 87-126 |
| Taurine | 106 (79-132) | 89 (78-92) | 136 (115-152) | < 0.001 | 0.06 | 60-90 |
| Tyrosine | 34 (31-39) | 44 (39-52) | 53 (47-58) | < 0.001 | 0.001 | 30-47 |
| L-Carnitine | 19 (13-21) | 27 (17-31) | 35 (14-43) | 0.09 | 0.06 | ND |
