# Supplementary material for: Domestic dogs maintain clinical, nutritional, and hematological health outcomes when fed a commercial plant-based diet for a year
Source: PLoS One. 2024 Apr 16;19(4):e0298942. doi: 10.1371/journal.pone.0298942 (PMC11020905; doi:10.1371/journal.pone.0298942)
Supplement: S4 Table — Values refer to median (minimum—maximum). (DOCX) [file pone.0298942.s004.docx]

**S4 TABLE.** Nutrient analysis of serum vitamin concentrations in dogs consuming meat-based diets (baseline) versus plant-based nutrition (6 and 12 months). Values refer to median (minimum - maximum).

| **Vitamin** | **Unit** | **Baseline** | **6 months** | **12 months** | **P-value**  **(Friedman)** | **P-value**  **(Wilcoxon)** | **Ref. Values**  **(MSU/TAMU)** |
| --- | --- | --- | --- | --- | --- | --- | --- |
| *Lipid-soluble* |  |  |  |  |  |  |  |
| Vitamin A | ng/mL | 734 (437-1,295) | 924 (600-1,292) | 979 (769-1,330) | 0.01 | 0.01 | 400-1,200 |
| Vitamin D | nmol/L | 120 (57-418) | 228 (91-383) | 257 (173-418) | < 0.001 | 0.004 | 109-423 |
| Vitamin E | ug/mL | 42 (21-74) | 31 (25-56) | 37 (25-57) | 0.62 | 0.33 | 4-12 |
| *Water-soluble* |  |  |  |  |  |  |  |
| Folate (B9) | ug/L | 9 (5-23) | 11 (4 -15) | 11 (4-37) | 0.04 | 0.09 | 8 – 24 |
| Cobalamin (B12) | ng/L | 364 (261-639) | 427 (310-619) | 426 (278-505) | 0.25 | 0.17 | 251 - 908 |
