# Supplementary material for: Domestic dogs maintain clinical, nutritional, and hematological health outcomes when fed a commercial plant-based diet for a year
Source: PLoS One. 2024 Apr 16;19(4):e0298942. doi: 10.1371/journal.pone.0298942 (PMC11020905; doi:10.1371/journal.pone.0298942)
Supplement: Attachment — Submitted filename: PLOS_RESPONSE TO REVIEWERS_12_16_23.docx [file pone.0298942.s006.docx]

REBUTTAL LETTER

RESPONSE TO ACADEMIC EDITOR & REVIEWER COMMENTS

12/16/23

PONE-D-23-18094R1

Domestic dogs maintain clinical, nutritional, and hematological health outcomes when fed a commercial plant-based diet for a year

PLOS ONE

Response to Reviewer #4

Reviewer #4: While I agree that the paper and findings support the use of plant-based diets as the main source of nutrition, there is still the possibility of alternative nutrition being available and uncontrollable, as well as a lack of a control group to compare to. The use of the baseline as each individual control is quite far removed from the end of the study (a year later) with limited ability to understand aging or seasonal effects to the parameters. 1 - 11 years is a big range on top of potential breed confounding factors.

We thank the reviewer for this important observation and agree that there are always inherent limitations of different study designs, including the one chosen for this study. The possibility of alternative nutrition is inevitable in a study that includes client-owned dogs living in households outside a restrictive laboratory environment. While one can focus on the limitations of work with client-owned dogs, there are also clear advantages. The study of laboratory beagles in a controlled environment does not reflect the true diversity of canine lifestyles and breeds within different home environments. We find the advantages of studying client-owned companion dogs far outweigh any concerns.

Having dogs serve as their own controls is considered a statistically sound approach.

If we had introduced a separate control group, we would have added more variation. The study did not attempt to understand effects of aging or seasonal changes and this was never within the scope. All adult dogs who were volunteered for the study were enrolled. It is correct that one could have elected a narrow range of age, body weight, breed, etc. However, this would also have meant that we would have had a much more difficult time enrolling a sufficient number of dogs in this study. In other words, enrolling dogs in a clinical study is also subject to logistic limitations such as budget and location.

STUDY DESIGN:

L99-101: Why is there reference to a sample timeframe of every 3 months if it was disregarded for the analysis? If the samples were taken, I feel it would be valuable to show the data, even if it is not analyzed in the final dataset.

As mentioned in lines 94-98, all dogs were examined quarterly, however, samples were collected at 0, 6, and 12 months. Consequently, we show data from three timepoints. Also, analyzing samples quarterly would have required a significantly larger budget.

RESULTS:

There is no mention of the questionnaire results. Due to their inclusion in the methods, this is a miss.

We have added the following (lines 213-214) in response to the comment: “No adverse effects were reported for any of the dogs in response to the eight questions included in the client telephone questionnaire.”

DISCUSSION:

This contains references to figures already described in the results (i.e. Fig 1, Fig 2 etc.) and feels repetitive. I don't believe there is a need to re-reference the figure references.

We appreciate the comment and understand that some readers might find this repetitive. Having said that, we have included the figure references to enhance readability for those who benefit from this approach and to improve accessibility.

L389: statement should include lack of analysis of Taurine in urine too.

Thank you for noting this omission. We have revised the text (line 379): “We did not measure taurine levels in whole blood or urine, which is a study limitation”

L441-444: mention of questionnaire again but no clarity on any results/findings from this. This section implies that the questionnaire would give clarity or reassurance on the compliance to the study protocol (i.e. no meat-based treats), however the questions do not cover additional feeding explicitly. I am concerned that the questionnaire is inadequate to aid in ensuring compliance.

We have added the following text (line 433): “During the phone interviews, it was moreover confirmed that all canine participants were fed only the prescribed plant-based study diet/treats (i.e., no foods or treats containing animal-based products).”
